# Identifying Core Affect in Individuals from fMRI Responses to Dynamic Naturalistic Audiovisual Stimuli

**DOI:** 10.1371/journal.pone.0161589

**Published:** 2016-09-06

**Authors:** Jongwan Kim, Jing Wang, Douglas H. Wedell, Svetlana V. Shinkareva

**Affiliations:** 1 Department of Psychology, University of South Carolina, Columbia, South Carolina, United States of America; 2 Department of Psychology, Carnegie Mellon University, Pittsburgh, Pennsylvania, United States of America; University of Würzburg, GERMANY

## Abstract

Recent research has demonstrated that affective states elicited by viewing pictures varying in valence and arousal are identifiable from whole brain activation patterns observed with functional magnetic resonance imaging (fMRI). Identification of affective states from more naturalistic stimuli has clinical relevance, but the feasibility of identifying these states on an individual trial basis from fMRI data elicited by dynamic multimodal stimuli is unclear. The goal of this study was to determine whether affective states can be similarly identified when participants view dynamic naturalistic audiovisual stimuli. Eleven participants viewed 5s audiovisual clips in a passive viewing task in the scanner. Valence and arousal for individual trials were identified both within and across participants based on distributed patterns of activity in areas selectively responsive to audiovisual naturalistic stimuli while controlling for lower level features of the stimuli. In addition, the brain regions identified by searchlight analyses to represent valence and arousal were consistent with previously identified regions associated with emotion processing. These findings extend previous results on the distributed representation of affect to multimodal dynamic stimuli.

## 1.0 Introduction

The two major components of core affect posited to underlie more complex emotions are valence, varying from negative to positive, and arousal, varying from low to high [1, 2]. Researchers have identified physiological correlates of valence and arousal across several different types of affect eliciting stimuli [3–5]. Similarly, neuroimaging studies have also demonstrated a number of correspondences between different neural activation patterns and levels of valence and arousal for typical [6–8] and clinical [9] populations. These results support the idea that the neural representations of valence and arousal should be identifiable on a trial-by-trial basis for a variety of different types of stimuli.

There has been growing interest in investigating the utility of multivariate pattern analysis (MVPA) approaches applied to fMRI data in clinical populations. MVPA is ideal in this regard because it is designed to analyze the data at the level of individual, which is critical in clinical applications. Much of the clinical work to date has examined functional responses to static affective stimuli, such as pictures of faces [10–12] or the images found in the International Affective Picture System (IAPS) [13]. There is a recent growing interest in affective processing of dynamic multimodal stimuli (see recent special issue, [14]) At the same time, it has been argued that naturalistic viewing conditions that are dynamic and multimodal, as well as low in task demands, may be critical in examining emotion perception in clinical populations [15]. For instance, a selective deficit to dynamic social stimuli, but not static stimuli, has been identified in individuals with autism spectrum disorders [16]. Impaired multisensory integration of emotions has been shown in schizophrenia [17] [18]. Moreover, context, that is more readily available in dynamic multimodal stimuli, has been shown to play an important role in emotion perception [19]. Therefore, it may be most relevant to examine affective responses to naturalistic multimodal stimuli that are closer to what is encountered in more realistic and natural settings, increasing the ecological validity of unimodal experimental designs [20, 21]. A first step in this direction is to establish that valence and arousal elicited by dynamic naturalistic stimuli can be reliably identified from fMRI data in a typical sample.

Previous fMRI studies have used MVPA to investigate the representation of affect, successfully decoding affective states from patterns of brain activity located in specific regions of interest as well as from patterns of whole brain activity [22]. However, most of those studies used static, visual stimuli and only a few studies investigated other modalities or dynamic stimuli [23], such as sounds [24, 25], smells [26], or autobiographic recall [27]. Thus it is unclear whether the affective dimensions of valence and arousal are identifiable from fMRI data using dynamic multimodal stimuli.

Behavioral studies have shown that multimodal presentation of congruent face and voice expressions facilitated emotion perception [28] compared to unimodal presentation. Context-rich multimodal stimuli may enhance affect recognition for patients with traumatic brain injury [29] and autism [30], but the reverse pattern was also reported for schizophrenic patients [31], possibly due to the failure of information integration across the two modalities. Brain activation evoked by natural dynamic stimuli has been shown to be highly reliable [32]. Behavioral studies of facial expressions have shown that dynamic stimuli are more easily recognized than static stimuli in both healthy populations [33, 34] and in clinical populations [35]. Neuroimaging studies have also found more extended activation patterns for dynamic facial expressions [36]. Many of the emotion studies comparing static versus dynamic conditions have utilized facial expressions, with few employing other types of stimuli [37]. Thus the representation of affective states in the brain induced by naturalistic dynamic stimuli requires further investigation.

Audiovisual clips may be a particularly relevant stimulus format to explore in linking experiences to more real world situations. In a constantly changing, dynamic environment, perceptual and affective components from multiple modalities tend to co-occur so that, for instance, one both sees and hears a laughing child on a playground. Audiovisual clips of affectively charged everyday occurrences present a dynamic and continuous audio-visual unfolding of events over time, more typical of naturalistic experience. These types of stimuli have a long history in emotion research [38, 39]. For example, film stimuli have been shown to produce differential psychophysiological response patterns to valence [40, 41]. Visual and auditory modalities that are stimulated together are more naturalistic in everyday life and may result in response enhancement and an advantage of redundancy [42]. Unlike static stimuli, audiovisual stimuli preserve natural timing relations and resolve ambiguities present in each separate modality [43].

One of the challenges of using naturalistic stimuli in research is the possible confounding of lower level features. For example, valence has been shown to be positively correlated with brightness [44] and level of arousal has been shown to be related to loudness and motion [45, 46]. Moreover, it has been demonstrated that affective states can be identified solely from lower level features. For example, using musical pieces, Coutinho and Cangelosi [47] identified arousal from lower level features such as loudness, tempo, pitch level, and sharpness, and they identified valence from features such as tempo and pitch level. In our experiment, we manipulate the affective states elicited by short videos along the dimensions of valence and arousal, producing four groupings of stimuli. We exercise some control of the semantic content of these audiovisual clips by distributing topics (human, animal, and inanimate) across the affective categories. To keep the stimuli naturalistic, we did not directly control the perceptual features, with an exception of sound intensity. Instead, we attempted to have a broad range of values of lower level features within each affective category and then control for these effects statistically and through the use of functional localizers. Thus, we sought to identify affective states under more naturalistic viewing conditions from neural patterns of activity measured with fMRI, after controlling for semantic and lower level features. By controlling for lower level features, we enhance the interpretation of our classification results as reflecting valence and arousal.

In summary, the goal of this study was to identify affective content of naturalistic audiovisual stimuli in individuals from fMRI data on a single trial basis, thus extending previous work that identified valence and/or arousal derived from static visual stimuli using MVPA methods based on fMRI data [7, 48]. In addition, we examine the neural representation of arousal and valence components of core affect in terms of areas that lead to identification of these core dimensions of affect. In this auxiliary analyses, we employ a decoding based searchlight analysis and compare the regions identified with those implicated in previous research as being important in emotion processing.

## 2.0 Method

### 2.1 Participants

This research was approved by the Institutional Review Board at the University of South Carolina. All volunteers gave written informed consent in accordance with the Institutional Review Board at the University of South Carolina. Eleven (five female, two left handed) volunteer adults (mean age 23.91 years, *SD* = 4.02) from the University of South Carolina community participated in the fMRI experiment. A separate group of volunteer adults (*n* = 49) from the same community participated in a preliminary behavioral experiment for stimulus validation. All participants in the fMRI experiment reported normal hearing, normal or corrected to normal vision, and no history of neurological diseases.

### *2.2* Materials

Participants viewed affect-eliciting audiovisual clips that varied on levels of valence and arousal. Naturalistic audiovisual stimuli were selected ad hoc from a larger in-house behaviorally-validated stimulus set, with the goal to maximize differences in valence for positive versus negative sets and maximize differences in arousal for low versus high arousal sets, while attempting to match levels on the shared dimensions across sets so that valence and arousal values are orthogonal. Eight stimuli were selected for each affective category corresponding to each of the four quadrants of the affective space: high arousal-negative valence (HN), low arousal-negative valence (LN), low arousal-positive valence (LP), and high arousal-positive valence (HP). The stimuli were balanced on semantic content across the affective categories. Each affective category included four clips with humans, two clips with animals, and two clips with inanimate content. The stimuli did not contain speech or written language. Some audio components contained prosodic information. All stimuli were selected to reflect a single topic with homogeneous affective content for the duration of the clip (e.g., children laughing and running around a playground).

**Fig 1:**
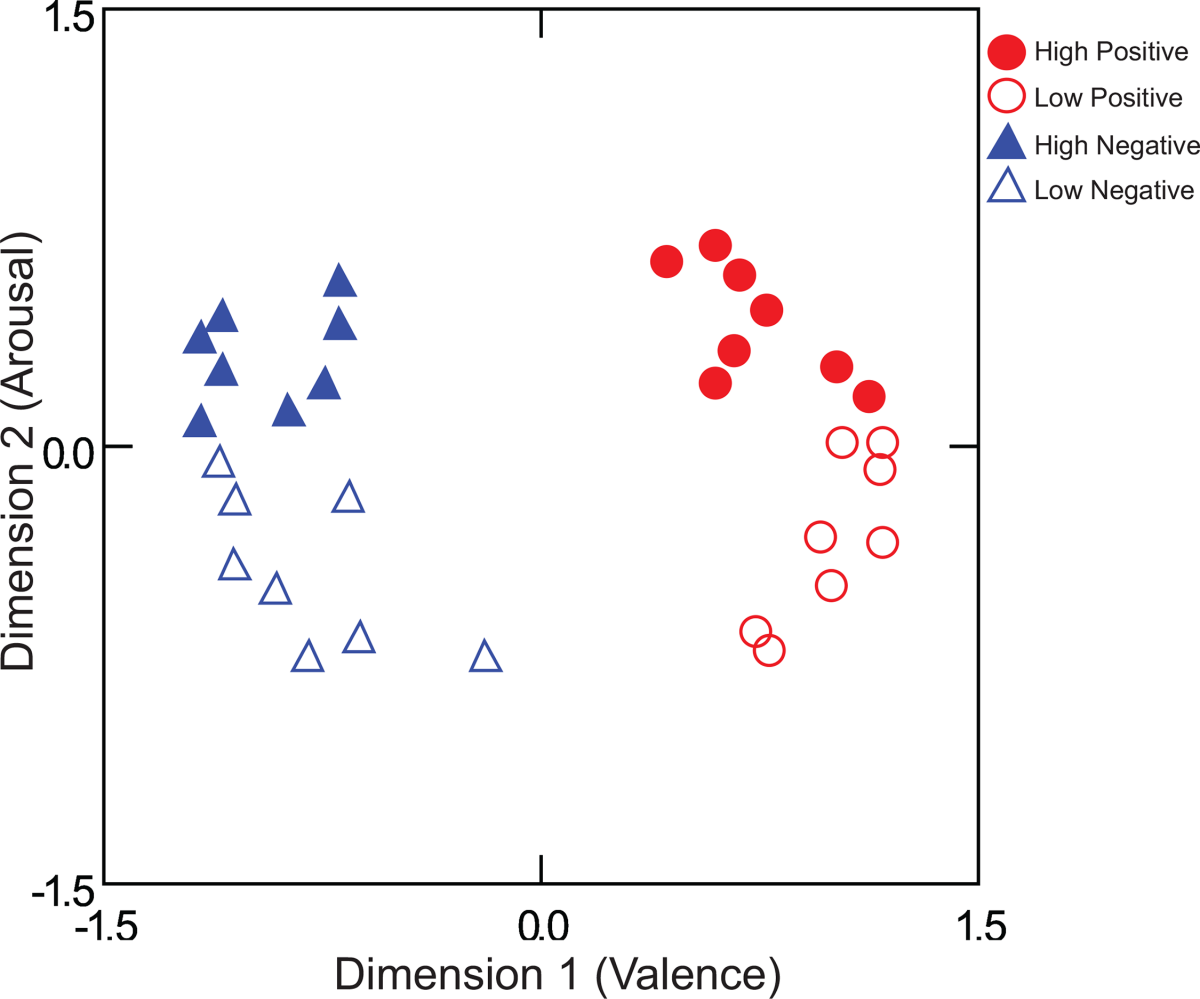
Lower dimensional representation of affective videos based on behavioral data. A two-dimensional solution from a separate group of participants described the data well (stress = .282, R^2^ = .543, *n* = 49).

The 32 selected audiovisual stimuli were validated in a behavioral experiment on a separate group of participants (*n* = 49). Affective categories of stimuli were shown to differ in a non-overlapping way on the dimensions of valence and arousal (Table 1 and S1 Table). Participants were asked to rate their emotional states along one of six dimensions after each video. The dimensions reflected the degree to which the participant reported feeling excited, positive, calm, anxious, negative, or sad. A correlation matrix of the 32 stimuli across the six ratings was constructed for each individual and the combined data was analyzed with INDSCAL [49]. The dimensions of valence and arousal naturally emerged from the ratings of the 6 dimensions, even though the two dimensions were not rated directly (Fig 1). This method was used to minimize bias in demand characteristics in responding that could arise from direct ratings of valence and arousal [50]. In an Analysis of Variance (ANOVA) conducted on the dimensional values from the MDS solution, the positive videos were significantly higher than the negative videos in mean valence configuration values, *F*(1,28) = 439.21, *p* < .001, *η*_*p*_^*2*^ = .969 (M_Positive_ = .87 and M_Negative_ = -.87) and the high arousal videos were significantly higher than the low arousal videos in mean arousal values, *F*(1,28) = 79.48, *p* < .001, *η*_*p*_^*2*^ = .739 (M_High_ = .37 and M_Low_ = -.37).

**Table 1:** Description of audiovisual stimuli. Means and standard deviations are shown.

Description	Affective Category				F-test	
	Negative Valence, High Arousal	Negative Valence, Low Arousal	Positive Valence, High Arousal	Positive Valence, Low Arousal	Valence	Arousal
Valence	-0.92 (0.24)	-0.83 (0.25)	0.78 (0.16)	0.96 (0.16)	*F*(1,28) = 583.87***	*ns*.
Arousal	0.37 (0.11)	-0.24 (0.27)	0.25 (0.18)	-0.38 (0.29)	*ns*.	*F*(1,28) = 62.17***
Hue	0.35 (0.12)	0.26 (0.11)	0.41 (0.18)	0.34 (0.24)	*ns*.	*ns*.
Saturation	0.25 (0.08)	0.27 (0.16)	0.33 (0.16)	0.35 (0.18)	*ns*.	*ns*.
Value (Brightness)	0.47 (0.09)	0.52 (0.07)	0.60 (0.12)	0.54 (0.14)	*ns*.	*ns*.
Amplitude (dB) (left)	10.00 (3.38)	14.75 (3.26)	13.78 (2.79)	11.85 (2.86)	*ns*.	*ns*.
Amplitude (dB) (right)	10.44 (3.55)	14.76 (3.26)	13.73 (2.80)	11.31 (2.60)	*ns*.	*ns*.
Frequency (Hz) (left)	379.22 (361.05)	241.26 (137.93)	465.58 (697.45)	1161.76 (1441.87)	*ns*.	*ns*.
Frequency (Hz) (right)	454.71 (372.69)	241.66 (137.70)	491.21 (687.18)	1170.00 (1435.29)	*ns*.	*ns*.
Motion 1 (slow and drifting)	122108.01 (54831.82)	36616.89 (34589.29)	91659.27 (44101.18)	32263.66 (31748.91)	*ns*.	*F*(1,28) = 23.469***
Motion 2	53812.82 (22459.94)	17790.62 (14413.37)	43402.15 (20830.68)	15407.53 (14798.81)	*ns*.	*F*(1,28) = 24.016***
Motion 3	38860.56 (15262.57)	14337.7 (10537.67)	30810.13 (14032)	12256.57 (11724.12)	*ns*.	*F*(1,28) = 21.884***
Motion 4	24938.01 (9014.75)	10388.2 (6386.2)	18924.16 (8638.21)	8335.32 (7803.32)	*ns*.	*F*(1,28) = 19.629***
Motion 5	15024.27 (4520.56)	6986.16 (3533.85)	12377.26 (5407.69)	5579.42 (4900.99)	*ns*.	*F*(1,28) = 20.431***
Motion 6	7305.06 (2337.75)	3863.75 (1697.84)	6830.85 (3381.41)	2720.06 (2723.28)	*ns*.	*F*(1,28) = 16.776***
Motion 7 (fast and transient)	2597.47 (846.05)	1308.79 (695.17)	2668.86 (2197.91)	989.22 (911.23)	*ns*.	*F*(1,28) = 10.275**

Note:

** p < .01

*** p < .001.

Hue, saturation, and value (brightness) were measured on 0 to 1 HSV scale; motion features were measured by the number of pixels of differences between frames.

Auditory components of the stimuli were normalized to the same mean amplitude by setting the values for each sound file extracted from the original video stimuli to the overall mean computed across all sound files using MATLAB (R2010b, MathWorks, Inc.). To keep the audiovisual stimuli naturalistic, no attempt was made to equate the stimuli on any other lower level features. The differences in lower level visual (hue, saturation, brightness, and motion) and four acoustical (bilateral frequency and amplitude) features across the affective categories were examined. Hue, saturation, and brightness of visual features were computed for each video by averaging the means of each frame of the video using the MATLAB *rgb2hsv* function. No significant differences in mean hue, saturation, or brightness values were found across valence or arousal conditions, *p*s >.05 (Table 1). Estimation of total motion for each video stimulus was based on absolute differences of pixels between frames at several time rates from slower or drifting motions to fast transient motions, without respect to the direction of motion that causes differences. Seven motion parameters were estimated at several time differences from slow drifting motions (e.g., walking) to fast transient motions (e.g., running). ANOVAs conducted on the total motion parameter for the 32 stimuli indicated that positive and negative videos differed in the total motion, *F*(7, 22) = 2.95, *p* =. 024, *η*_*p*_^*2*^ = .484, and high and low arousal videos differed in the total motion, *F*(7, 22) = 4.474, *p* = .003, *η*_*p*_^*2*^ = .587. Negative videos had greater total motion than positive videos and high arousal videos had greater total motion than low arousal videos. Separate ANOVAs conducted on each constituent motion parameter failed to find any significant effects of valence (*p* > .05), although all seven indicated significant effects of arousal (*p*s < .001). Finally, frequency and amplitude for left and right channels were measured with Frequency Analysis and Amplitude Statistics functions of Adobe Audition CS6. The four experimental sets did not differ from each other in mean frequency or amplitude, *p*s > .05.

### *2.3* fMRI acquisition

MRI data were acquired on a Siemens Magnetom Trio 3.0T whole-body scanner (Siemens, Erlangen, Germany) at the McCausland Center for Brain Imaging at the University of South Carolina. The functional images were acquired using a single-shot echo-planar imaging pulse sequence (TR = 2200ms, TE = 35ms, 90° flip angle) with a 12-channel head coil. Thirty-six 3 mm thick oblique-axial slices were imaged in interleaved scanning order with no gap. The acquisition matrix was 64×64 with 3×3×3 mm voxels. Functional data was acquired using a slow event-related design in two scanning sessions (two runs for each session). High-resolution whole-brain anatomical images were acquired using a standard T1-weighted 3D MP-RAGE protocol (TR = 2250 ms, TE = 4.18 ms, FOV = 256 mm, flip angle = 9°, voxel size = 1×1×1 mm) to facilitate normalization of the functional data.

### *2.4* Functional Localizer

**Fig 2:**
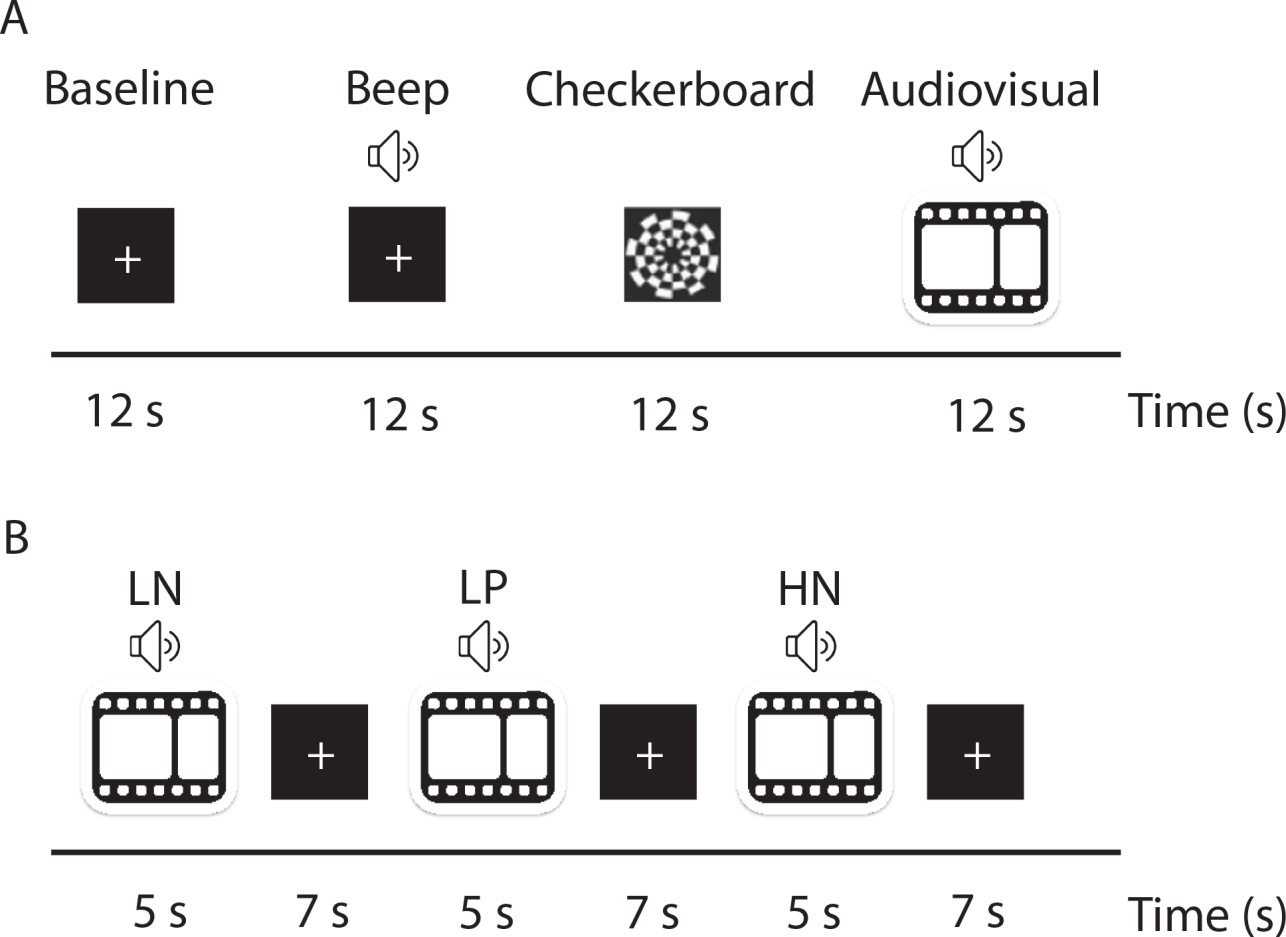
A schematic representation of the presentation timing. (A) Functional localizer. Participants were presented with baseline, auditory (beep), dynamic visual (checkerboard), and naturalistic audiovisual stimuli in a block design. Each block lasted for 12s. (B) Main experiment. Participants were presented with naturalistic audiovisual stimuli selected from the four quadrants of the affective space: high arousal negative valence (HN), low arousal negative valence (LN), low arousal positive valence (LP), and high arousal positive valence (HP). Each audiovisual clip was presented for 5s, followed by 7s fixation.

Areas responsive to naturalistic audiovisual presentation were identified in a separate localizer session for each participant. There were four experimental conditions: baseline, auditory (beep), dynamic visual (checkerboard), and a combined naturalistic audiovisual condition (audiovisual). Additionally, the functional localizer contained two other conditions that were not part of the present experiment. The conditions were presented in a block design with each block lasting 12s (Fig 2A). Baseline condition consisted of a black screen with a white fixation cross shown in the center of the screen and background noise. During an auditory condition, designed to localize primary auditory cortex, a binaural sine tone of 1,000 Hz pulsating at 6 Hz was presented [51]. During a dynamic visual condition, designed to localize primary visual areas, there were 12s of high-contrast flickering checkerboard reversals, 200ms per cycle [52]. During a naturalistic audiovisual condition, four 3s audiovisual clips sampled from the four affective quadrants were played back to back. By including a wide range of affective conditions the localizer is not sensitive to facets of the stimuli that discriminate between affective conditions. There were eight runs in the localizer scan and each run consisted of six conditions presented in a pseudo randomized order such that the same condition was not presented twice in a row for the two subsequent runs. There were a total of 48 blocks resulting in 273 acquired volumes. All stimuli were different from those presented in the main experiment. Audio-visual clips used for the localizer scan were not repeated within the localizer scan.

### 2.5 Experimental paradigm

Functional magnetic resonance imaging was used to measure brain activity while participants were presented with affect-eliciting audiovisual clips. All stimuli were presented using E-prime software (Psychology Software Tools, Sharpsburg, PA). Audiovisual stimuli were presented in 320 × 240 pixels resolution in 32-bit color at a rate of 25 frames per second onto a 640 × 480 resolution screen. Sound was delivered via Serene Sound Audio System (Resonance Technology Inc, Northridge, CA). Each clip was presented for 5s, followed by a white fixation cross shown on black background for 7s (Fig 2B). Participants were instructed to focus on the fixation cross in the center of the screen throughout the experiment. For each participant, 128 trials were presented in 4 blocks of the 32 unique exemplars. Within each block, the 32 exemplars were randomly presented with the restriction that no stimuli from the same affective condition were presented twice in a row and that each affective state was presented within each successive block of four trials.

### 2.6 fMRI data processing and analysis

Data processing and statistical analyses were performed in MATLAB environment using standard procedures in Statistical Parametric Mapping software (SPM 8, Wellcome Department of Cognitive Neurology, London, UK). The data were corrected for motion and linear trend. Structural data were segmented into white and gray matter to facilitate the normalization. Functional and anatomical images were co-registered and spatially normalized into the standard Montreal Neurological Institute (MNI) space based on T1-derived normalization parameters.

*Functional localizer*: For each participant we have identified voxels that were more responsive to audiovisual condition compared to baseline (VA) (*p* < .05, FWE-corrected, cluster size > 5), but excluding those voxels that were more responsive to checkerboard condition compared to baseline (VP) (*p* < .05, FWE-corrected, cluster size > 5) and those voxels that were more responsive to beep condition compared to baseline (AP) (*p* < .05, FWE-corrected, cluster size > 5).

*Main experiment*: To improve signal-to-noise ratio, the time-series data for each voxel were fit using *GLMdenoise* [53], a technique successfully used in MVPA and other applications [54, 55]. *GLMdenoise* used the four affective categories to estimate regressors of no interest. Notably the procedure was blind to valence and arousal categories, and thus did not bias the results when comparing across combined categories (i.e., positive versus negative valence or high versus low arousal). Furthermore, to control for possible confounds arising from lower level features of the stimuli, five lower level feature components were regressed out as covariates of no interest together with six head motion parameter estimates. We used principal components to reduce the number of regressors, while keeping most of the variability in the data. The five lower level feature components were generated from three separate principal components analyses: two scores from three visual features which captured 81.91% of the variance for the visual features, one score from seven motion features (88.37%), and two scores from four auditory features (99.03%). The residuals from this analysis were used for all further analyses. The percent signal change (PSC) relative to the average activity in a voxel was computed for each voxel in every volume from the residuals. The mean PSC of two volumes, offset 4.4s from the stimulus onset (to account for the delay in hemodynamic response), was used as the input for further analyses [7]. Data for each condition were standardized across voxels to have zero mean and unit variance [56].

### 2.7 Multivariate Pattern Analyses

The MVPA methods employed in this work are similar to those that have been successfully used in our other exploration of affective space [7, 57]. Logistic regression classifiers [58] were trained to identify valence and arousal patterns of brain activity associated with affect-eliciting audiovisual stimuli. Two-way classifications were performed to identify valence (positive vs. negative) trials, as well as arousal (high vs. low) trials.

Within-participant classification was performed within functionally localized gray matter voxels that selectively responded to naturalistic audiovisual stimuli. In each of the four cross-validation folds, one presentation of the 32 exemplars was left out as test data when the classifiers were trained on the other three presentations. Prior to classification, trials were divided into training and test sets. The classifier was constructed from the training set and applied subsequently to the unused test set. Classification accuracies were computed based on the average classification accuracy across the four cross-validation folds. Furthermore, to test the generalizability of the affect representation across stimuli, we trained a valence classifier and an arousal classifier on 31 exemplars, and tested on the left out exemplar by decoding its valence or arousal. Each of the 32 exemplars was left out once for testing in a cross-validation fold. The average classification accuracy for the four presentations of the test exemplars was reported.

Cross-participant classification performance was evaluated with eleven-fold cross-validation, where data from one of the participants was left out for testing in each fold. The classifier was trained on data from all but one participant and used to make predictions for individual trial data from the left-out participant. Average classification accuracy across trials was computed as a measure of how well individual affective states can be identified based on data from other individuals. This procedure was repeated for all participants. Classifications were performed using all voxels as well as the union of functional localizer masks.

Statistical significance for the classification accuracies was evaluated by comparison to an empirically derived null distribution constructed by 1,000 non-informative permutations of labels in the training set. Classification accuracies with p-values smaller than .05 were considered significant.

### 2.8 Searchlight analyses

Searchlight analyses [59] were performed to localize regions that were sensitive to valence and arousal information. Searchlight analyses employ a sliding neighborhood with a predefined search radius to scan an entire volume. A union of areas sensitive to checkerboard and beep sounds (compared to baseline) were excluded from the subsequent searchlight analyses for each participant.

For each participant and each voxel, data from a 5×5×5 voxels neighborhood, centered at a given voxel, was extracted and used for the same MVPA procedure described above. The average classification accuracy was assigned to the center voxel. Two-way classifications for valence (positive vs. negative) and arousal (high vs. low) were performed across the gray matter voxels in the mask described above. Thus two classification accuracy maps were generated for each individual. Chance-level accuracy (.5) was subtracted from obtained classification accuracy maps [60] before they were submitted to a random-effects whole-brain group analyses.

Permutation tests were performed to find the empirically significant cluster sizes [61]. For each run, the same searchlight procedure was conducted as described above, but with a random permutation of condition labels for each individual. The individual accuracy maps generated using a random permutation of condition labels were submitted to a group analysis and the largest cluster size was recorded. This entire procedure was repeated 1,000 times each for valence and arousal classification (which are equivalent), yielding null distributions of cluster sizes.

Finally, additional confirmatory analyses were performed to verify that the identified clusters were sensitive to valence and arousal information [62]. The significant clusters found by searchlight analyses were based on group analysis, so that not all of the voxels within each searchlight cluster may be informative and represent affective states at the individual level. Thus an additional feature selection procedure was performed for each individual to exclude voxels that were not related to affective states. In the first confirmatory analysis, a within-individual classification analysis was performed for each cluster identified by the searchlight analysis. Prior to classification, a measure of stability was computed for all voxels within each searchlight by computing correlations across three folds in the training set. The top 80% of the most stable voxels from the training set were used for subsequent classification for each fold. Classification accuracies across the four cross-validation folds were averaged for each participant. Significance testing was conducted with a one-sample t-test to evaluate if the group mean accuracy was significantly above chance (.5). In the second confirmatory analysis, lower dimensional representation analyses were performed for each cluster identified by the searchlight analyses. These analyses used the top 80% of stable voxels. STATIS [63], a generalization of principal components analysis for multiple similarity matrices, was conducted to visualize the underlying structure of the exemplars within each searchlight cluster. This technique is based on the cross-product matrix, thus allowing the number of voxels in the analysis to vary across individuals [64]. Point-biserial correlations (r_pb_) between design values (1 for positive or high arousal, and -1 for negative or low arousal) and corresponding coordinates from the STATIS solutions were computed to evaluate if the solutions were indeed sensitive to valence information from valence clusters and arousal information from arousal clusters.

## 3.0 Results

### 3.1 Functional localizer

For each participant we have identified functional masks for voxels that were more responsive to audiovisual condition compared to baseline but excluding those voxels that were more responsive to checkerboard condition compared to baseline and those voxels that were more responsive to beep (VA∩(VP⋃AP)^c^). The number of voxels in each mask identified for each participant is shown in Table 2. For a concise summary, the functional localizer results are presented at a group level in S2 Table. Please note, individual masks (S1 Fig) were used for subsequent MVPA analyses; group localizer results are presented to facilitate comparison to other studies.

**Table 2:** Number of voxels for each of the masks reported by participant.

Participant	Gray matter	VA	VP	AP	VA∩(VP⋃AP)^c^	GM∩(VA∩(VP⋃AP)^c^)
1	12243	15554	7475	577	8864	2551
2	20279	11723	4006	682	8066	3086
3	24024	12255	15253	1286	4031	2040
4	12544	13294	6664	5193	6590	1261
5	16064	8926	3028	1008	5855	1977
6	15931	11947	10337	3298	3195	1031
7	17528	5221	1784	185	3449	1207
8	15766	8078	3372	538	5039	1351
9	21940	7558	1949	2048	5032	2142
10	8689	9935	3504	433	6810	1101
11	20825	13872	9461	1202	6674	2951

VA, voxels that were more responsive to audiovisual condition compared to baseline (*p* < .05, FWE-corrected, cluster size > 5); VP, voxels that were more responsive to checkerboard condition compared to baseline (*p* < .05, FWE-corrected, cluster size > 5); AP, voxels that were more responsive to beep condition compared to baseline (*p* < .05, FWE -corrected, cluster size > 5); VA∩(VP⋃AP)^c^: voxels that were more responsive to audiovisual condition compared to baseline (*p* < .05, FWE-corrected, cluster size > 5), but excluding those voxels that were more responsive to checkerboard condition compared to baseline (*p* < .05, FWE-corrected, cluster size > 5) and those voxels that were more responsive to beep condition compared to baseline (*p* < .05, FWE -corrected, cluster size > 5); GM∩(VA∩(VP⋃AP)^c^), the intersection between individual gray matter mask and VA∩(VP⋃AP)^c^. Participants are ordered by within-participant classification performance (see below).

### 3.2 Identification of valence

**Fig 3:**
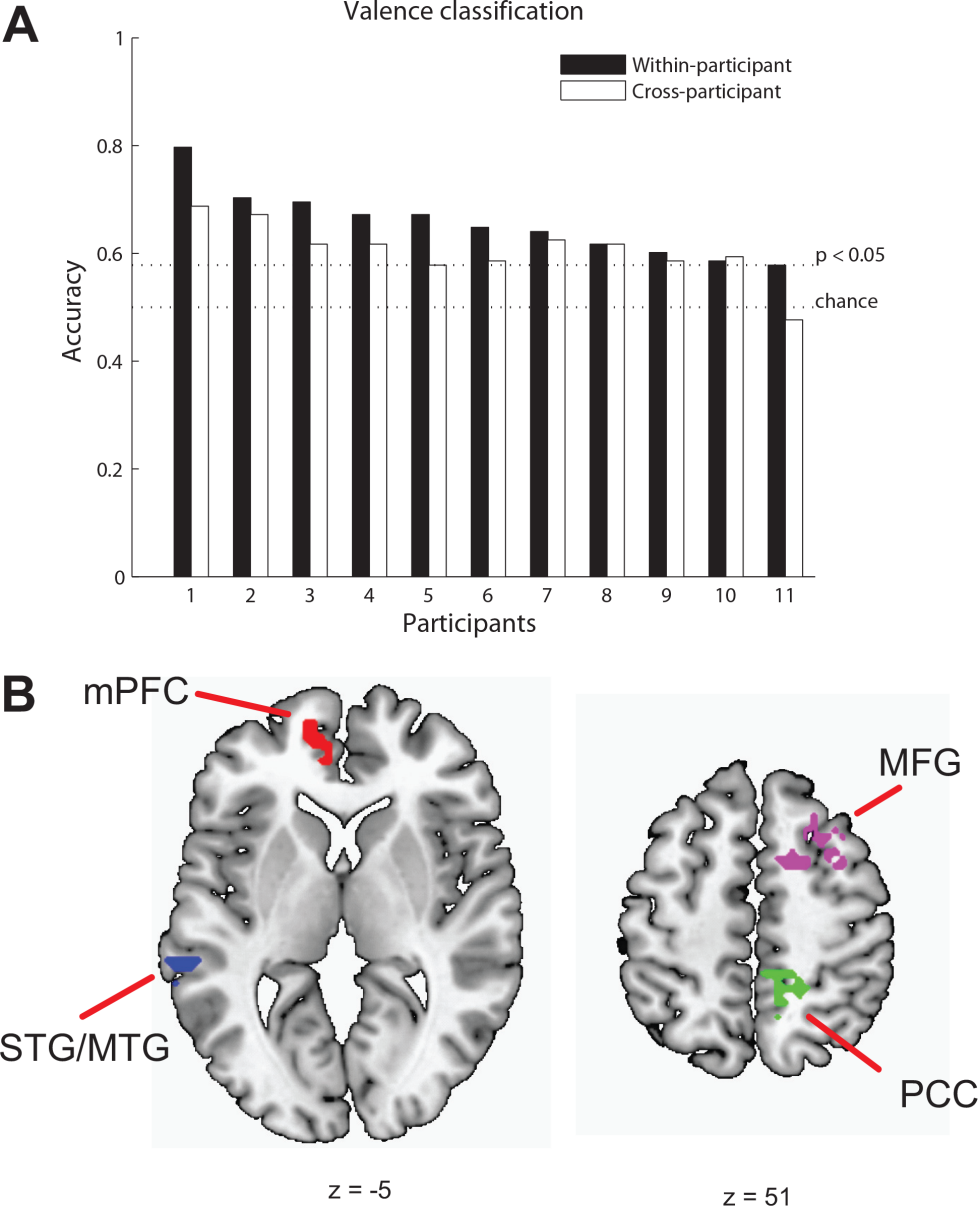
MVPA results for valence. (A) Classification accuracies for within-participant (filled bars) and cross-participants (unfilled bars) valence identification. Participants are ordered by within-participant classification performance. (B) Four clusters; the left medial prefrontal cortex (mPFC), the right posterior part of the cingulate cortex (PCC), the left superior/middle temporal gyrus (STG/MTG), and middle frontal gyrus (MFG) are shown on axial slices.

We examined whether the valence of the stimuli (positive or negative) could be identified for individual trials based on activity patterns elicited by viewing audiovisual clips, defined by the quadrants of the valence-arousal space. First, classifiers were trained for each participant to identify the valence category of the stimuli they were watching: positive or negative. Significant classification accuracies (*p* < .05) were found for ten of 11 participants (Fig 3A). Classification accuracies for eleven participants ranged from .59 to .80, with the mean of the accuracies (*M* = 0.66, *SD* = 0.06) significantly greater than chance, *t*(10) = 8.17, *p* < .001. Moreover, the valence states of audiovisual clips that were previously unseen by the classifier were identified with accuracy reliably above chance for nine out of 11 participants (cross-exemplar decoding, *M* = .74, *SD* = .13, range = (0.56, 0.94)), suggesting that it was the neural representation of valence, rather than individual stimuli-specific properties that drove the decoding accuracy.

To examine the similarity of valence representation across participants and to show the typicality of the decoding results we have used data from all but one participant to train a classification model and predict valence in the left out participant at the whole brain level of analysis. From all voxels, we were able to identify valence in nine of 11 participants with accuracies above chance, *p* < .05 (Fig 3A). Classification accuracies ranged from 0.48 to 0.69, with the mean of the accuracies (*M* = .61, *SD* = .06) significantly greater than chance, *t*(10) = 6.34, *p* < .001. To link these results more directly to the results for decoding within individuals, we restricted the analyses to the union of functional localizer masks. Under this restriction, we were able to identify valence in six out of 11 participants with accuracies above chance, *p* < .05. Classification accuracies ranged from .51 to .65, with the mean of the accuracies (*M* = .60, *SD* = .05) significantly greater than chance, *t*(10) = 7.05, *p* < .001.

Having established the typicality of the decoding results with cross-participant identification of valence, searchlight analyses were performed to spatially localize brain regions that were sensitive to valence. These revealed five clusters: the left medial prefrontal cortex (mPFC), the right posterior part of the cingulate cortex (PCC), the left superior/middle temporal gyrus (STG/MTG), the thalamus, and the middle frontal gyrus (MFG) (*p* < .05, cluster size > 43).

Confirmatory MVPA and STATIS analyses were performed within each cluster to verify the information content of identified clusters. The results of MVPA showed four out of five valence clusters (with mean accuracies of .60, .57, .56, and .54) significantly discriminated valence information, *p*s < .05 (Fig 3B; Table 3; S2 Fig). The classification accuracy from the thalamus cluster did not reach significance (*M* = .53, *p* = .14). The lower dimensional representation of 32 exemplars from the four valence clusters confirmed that each of the clusters identified by searchlight was informative of valence. The point-biserial correlations between design values and component values corresponding to valence for the four regions were .41, .46, .47, and .55, *p*s < .05 (also see S3 Fig). In sum, four clusters sensitive to valence (PCC, MFG, STG/MTG, and mPFC) were identified by searchlight analyses (Fig 3B, Table 3).

**Table 3:** Searchlight results for valence and arousal.

			MNI coordinates				
Anatomical region	Hemisphere	Cluster size	x	y	z	T	Z
Valence							
PCC	R	109	6	-46	31	8.89	4.58
MFG	R	87	30	11	52	7.57	4.28
STG/MTG	L	46	-63	-58	10	8.23	4.44
mPFC	L	44	-15	56	1	9.25	4.66
Arousal							
PC	R	56	9	-49	52	6.67	4.03
OFC	R	54	27	62	-17	7.47	4.25

Note: *p* < .001, uncorrected, cluster size > 40 for valence and 50 for arousal. R, right; L, left; cluster size reported in voxels; T indicates peak t values; Z indicates peak z values; OFC: anterior part of orbitofrontal cortex; PC: precuneus; mPFC: medial prefrontal cortex; PCC: posterior part of the cingulate cortex; STG/MTG: superior/middle temporal gyrus; MFG: middle frontal gyrus.

### 3.3 Identification of arousal

**Fig 4:**
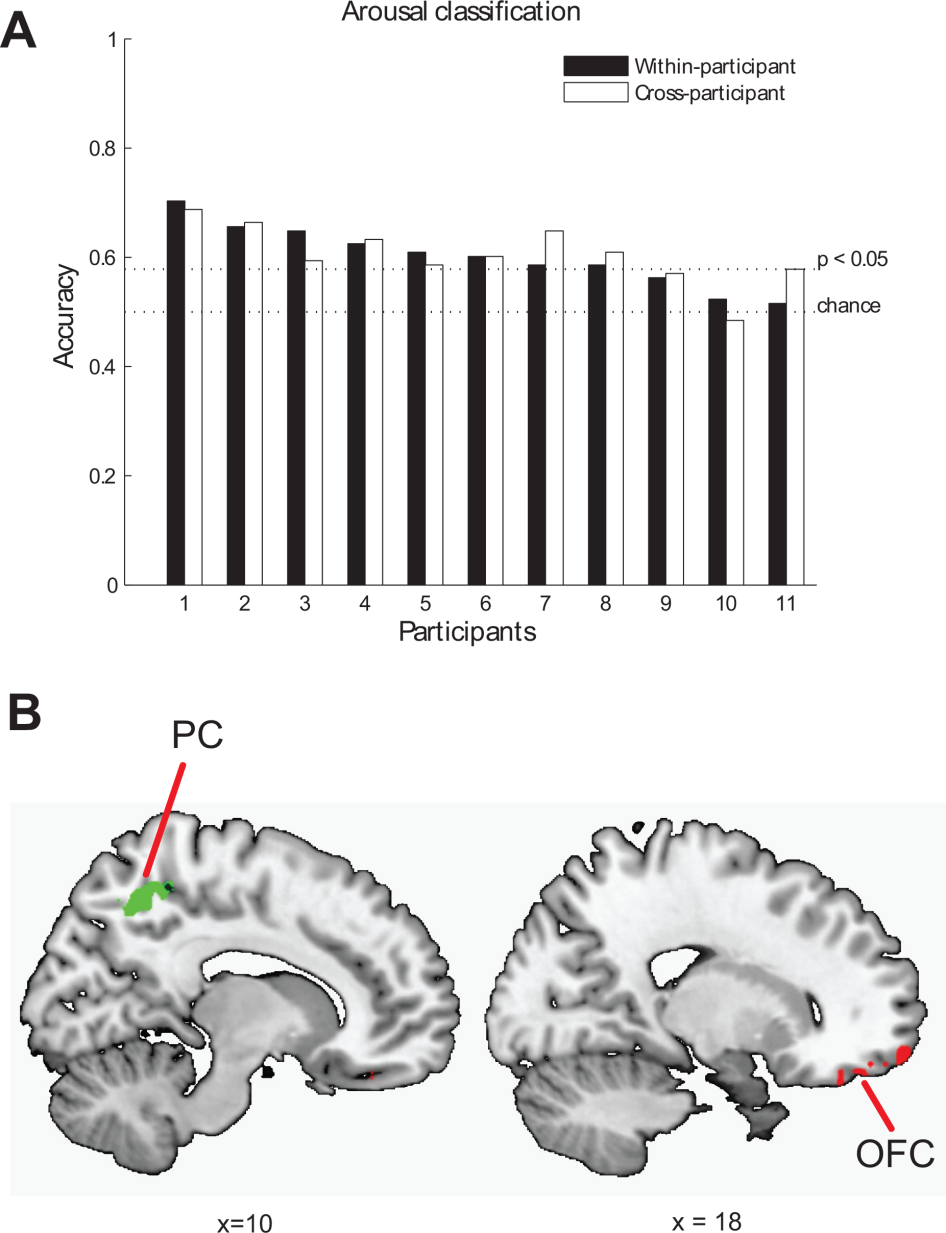
MVPA results for arousal. (A) Classification accuracies for within-participant (filled bars) and across-participants (unfilled bars) arousal identification. (B) Two clusters, the right anterior part of orbitofrontal cortex (OFC) and the right precuneus (PC) are shown on sagittal slices.

We examined whether the arousal of the stimuli (low or high) could be identified for individual trials based on activity patterns elicited by viewing audiovisual clips, defined by the quadrants of the valence-arousal space. Classifiers were trained for each participant to identify the arousal category of the audiovisual stimuli: high or low. Significant classification accuracies (*p* < .05) were found for eight of 11 participants (Fig 4A). Classification accuracies based on functionally defined ROI for eleven participants ranged from 0.52 to 0.70, with the mean of the accuracies (*M* = .60, *SD* = .06) significantly greater than chance, *t*(10) = 6.00, *p* < .001. The arousal states elicited by audiovisual clips that were previously unseen by the classifier were identified with reliably above-chance accuracy for nine out of 11 participants (M = .66, SD = .09, range = (0.50, 0.81)), suggesting that it was the neural representation of arousal, rather than individual stimuli-specific properties that drove the decoding accuracy.

To examine the similarity of arousal representation across participants and show the typicality of the decoding results we used data from all but one participant to train a classification model and predict arousal, high or low, in the left out participant. From all voxels, we were able to identify arousal in eight of 11 participants with accuracies above chance. Classification accuracies ranged from .48 to .69, with the mean of the accuracies (*M* = .61, *SD* = .05) significantly greater than chance, *t*(10) = 6.37, *p* < .001. To link these results more directly to the results for decoding within individuals, we restricted the analyses to the union of functional localizer masks. Under this restriction, we were able to identify arousal in six out of 11 participants with accuracies above chance, *p* < .05. Classification accuracies ranged from .43 to .64, with the mean of the accuracies (*M* = .58, *SD* = .06) significantly greater than chance, *t*(10) = 4.29, *p* < .01.

Having established the typicality of the decoding results with cross-participant identification of arousal, searchlight analyses were performed to spatially localize brain regions that were sensitive to arousal. Searchlight analyses revealed two significant clusters: the right precuneus (PC) and the right orbitofrontal cortex (OFC) (*p* < .05, cluster size > 50). Confirmatory MVPA and STATIS analyses were performed within each cluster to verify the information content of identified clusters. MVPA within each of the identified arousal clusters confirmed that mean classification accuracies (*M* = .56 and *M* = .57) from each cluster were significantly higher than chance, *p*s < .01 (Fig 4B; Table 3; S2 Fig). The lower dimensional representation confirmed that each of the clusters identified by searchlight was sensitive to arousal (S3 Fig). The point-biserial correlations between design values and component values corresponding to arousal for the two regions were indicative of the predicted relationship (OFC: *r*_*pb*_ = .33, *p* = .07, PC: *r*_*pb*_ = .59, *p* < .001). In sum, two regions sensitive to arousal (OFC and PC) were identified by the searchlight analyses (Fig 4B, Table 3).

## 4.0 Discussion

This study investigated how valence and arousal elicited by naturalistic multimodal stimuli are represented in the brain. We were able to identify valence and arousal for individual trials based on activity patterns elicited by the audiovisual stimuli for most of the individuals. This result was achieved when controlling for lower level features of the stimuli by excluding brain regions associated with lower level perceptual processing and removing the effects of lower level stimulus parameters statistically. By doing so we bolster the inference that identification of affective states was indeed attributable to valence and arousal. The fact that the neural representation of affect was conveyed by brain areas selectively responsive to naturalistic dynamic multisensory information is consistent with the idea that valence and arousal are two fundamental properties that are readily accessed when processing dynamic multimodal stimuli.

In addition to classifying valence and arousal within individuals, we also found that the affective states of an individual could be reliably modeled by training the classifiers on data from other individuals. This cross-participant classification implies common areas of representation of affective states across individuals. As in the case of within-participant classification, the cross-participant classification was based on voxel activity that controlled for similarity of lower level perceptual features across stimuli, supporting the conclusion that these activation patterns reflect the core dimensions of affect and not just perceptual regularities within affective categories. We used searchlight techniques to visualize spatially localized regions containing valence or arousal information. The areas identified by searchlight as sensitive to valence (PCC, MFG, STG, MTG, mPFC) and arousal (PC and OFC) information were consistent with the areas previously implicated in emotion processing.

The regions found to be involved in valence in the current study were PCC, MFG, STG/MTG, and mPFC. It has been reported that the PCC is activated in emotional word processing [65–67], representation of associated emotions [60], and modality-general representation of complex emotional information [68]. The STG and MTG have been implicated in integration of multimodal affective information [28, 69–72], and more generally, in integration of multimodal (mostly visual and auditory) sensory information [73]. However, it is not clear whether valence, arousal or both are represented in this region, because most of the aforementioned studies utilized discrete emotions (e.g., happy, disgusted, or angry). While the current study provides support for valence related processing in these regions, more work is needed to determine if arousal is represented in these regions. The mPFC is often found to be involved in processing of emotion [74]. It has been implicated in the perception of affect in faces and scenes [75]. The mPFC has been shown to be related to consistent representation of discrete emotional states from face, voice, and body movement, suggesting modality-general processing of emotion within the mPFC [70]. The current study found the mPFC is sensitive to valence, suggesting that valence might be driving this distinction.

The regions found to be involved in arousal in the current study were PC and OFC. The OFC has been reported to be engaged in processing of affective word stimuli [76], odors [77] and tastes [78]. It has been identified as representing valence for both pictures and tastes, i.e., a modality-general representation of affect [78]. In our study, the OFC was identified to be sensitive to arousal information, which is another dimension of core affect (the cluster size within OFC for valence fell at the top 7.4% within the null distribution, which was not statistically significant). These findings, taken together, implicate the OFC in general affective processing.

In sum, the regions identified as sensitive to valence and arousal information in the current study are consistent with the regions identified in literature linked to emotion. Moreover, many of six regions have been linked to modality-general representations of emotion [68, 70, 78]. Our findings are consistent with the idea that the core affect dimensions of valence and arousal may underlie the processing of emotions in these regions for multimodal affective stimuli. The clusters that we have shown to be sensitive to valence and arousal in this study are consistent with the idea that these areas may contribute to the brain’s affective workspace [79].

Past research has demonstrated that modality-congruent sensory areas are also involved in affect processing. For example, patterns of activity in voice-sensitive cortices were found to represent categorical emotional vocal expressions [80], while activity patterns in fusiform face area represent facial expressions [81]. Similarly, Lang, Bradley [82] presented emotion-inducing pictures and found a greater functional activity for emotional pictures than for neutral pictures in primary visual regions, including occipital gyrus, fusiform gyrus, and lingual gyrus. Both valence and arousal have been demonstrated to be represented in the visual cortex [83]. Thus, successful classifications of valence and arousal are expected in the modality-congruent regions. We excluded sensory regions that were localized functionally to minimize the effect of the lower level features and thereby enhance interpretation of our classification in terms of affective features rather than sensory features.

In developing a stimulus set that independently manipulated valence and arousal, we chose to exclude the neutral valence condition. This is because neutral valence conditions are difficult to match on arousal levels with corresponding positive and negative valence conditions. Though two core affect dimensions, valence and arousal, are assumed to be independent, unsigned valence dimension (positive/negative vs. neutral) may relate to arousal. For example, affective stimuli data sets such as the International Affective Picture System (IAPS) and International Affective Digitized Sounds (IADS) databases [84, 85] show distributions of U-shaped pattern, indicating valenced stimuli tend to be more arousing compared to neutral stimuli. Thus, it is difficult to find stimuli that are neutral with moderate or high arousal level and it is more likely for neutral stimuli to be associated with less arousal level compared to valenced stimuli. For example, [86] found brain regions that activated as quadratic function of valence raising a possibility that these results might be confounded with arousal dimension. Consistent with this idea, Viinikainen, Kätsyri [87] reported extreme positive and negative valence stimuli were associated with high arousal. The relationship between valence and arousal is still open for the further investigation.

The results of our study have important implications for affective processing in clinical populations. First, we used a passive viewing paradigm to successfully decode valence and arousal. This type of task may be more amenable to clinical populations, as it has been argued that implicit tasks may reduce task demands. Explicit tasks, such as emotion categorization or matching of emotional stimuli, can be problematic in clinical populations that may suffer from executive dysfunction [15]. Second, the present study extends previous work by demonstrating that the affective dimensions of valence and arousal can be identified from dynamic audio-visual stimuli. Given the distinction between processing of affect from static and dynamic stimuli for some clinical populations noted earlier [16], these representational analyses may be helpful in better understanding affective disorders. Third, the cross-participant classification utilized in the current study provides a basis for future classification studies of clinical populations based on a match to specific profiles for affective representation [13].

Several design facets limit the generalizability of the findings. First, we tried to make the stimuli as homogeneous as possible for the duration of the clip with an assumption of constant affective response to a single stimulus, but the methods in the current study have not been demonstrated for more transient nature of affective states in truly naturalistic settings. Future research may examine applications to longer and more variable stimuli. Second, this study is based on categorical affective states rather than continuous ones due to the difficulty of collecting neutral stimuli with varying arousal levels. As discussed above, it is challenging to manipulate neutral condition with moderate and high arousal levels. Finally, our results are correlational in nature; although we have shown that BOLD data contain enough information to identify the affective category of the stimuli, no inference can be made on how the brain uses this information.

In conclusion, we were able to identify valence and arousal states in individuals for single trials of multimodal dynamic stimuli while controlling for lower level features statistically and with functional localizers. We were able to do this both within and across individuals. The spatially localized areas found to be sensitive to valence and arousal information were consistent with the literature on affective states. These findings extend previous results on affective representation [7, 13] to naturalistic dynamic stimuli and identify possible brain regions for encoding valence and arousal representations.

## Supporting Information

S1 Fig Functional localizer masks for each of the participants. Voxels that were more responsive to audiovisual condition compared to baseline (*p* < .05, FWE-corrected, cluster size > 5), but excluding those voxels that were more responsive to checkerboard condition compared to baseline (*p* < .05, FWE-corrected, cluster size > 5) and those voxels that were more responsive to beep condition compared to baseline (*p* < .05, FWE-corrected, cluster size > 5) are shown in red.
(EPS)

S2 Fig **Within-participant classification accuracies obtained in confirmatory analyses for valence (left panel) and arousal (right panel).** Classification accuracies are summarized by box plots across the 11 participants for each cluster identified by searchlight analyses.
(EPS)

S3 Fig **STATIS solutions performed as confirmatory analyses within each searchlight cluster for valence (left panel) and arousal (right panel).** r_pb_ denotes point-biserial correlation coefficient between design values and component 1 coordinates, † *p* < .1, * *p* < .05, ** *p* < .01, *** *p* < .001.
(EPS)

S1 Table Description of 32 stimuli from the norming study (*n* = 49). (DOCX)

S2 Table Group summary of functional localizer masks. (DOCX)
