# Supplementary material for: Identifying Core Affect in Individuals from fMRI Responses to Dynamic Naturalistic Audiovisual Stimuli
Source: PLoS One. 2016 Sep 6;11(9):e0161589. doi: 10.1371/journal.pone.0161589 (PMC5012606; doi:10.1371/journal.pone.0161589)
Supplement: S1 Table — (DOCX) [file pone.0161589.s004.docx]

**Table S1. Description of 32 stimuli from the norming study (*n* = 49).**

| ID | Valence | Arousal | Description | Semantic Category | Valence | Arousal |
| --- | --- | --- | --- | --- | --- | --- |
| 1 | Positive | High | Dolphin Singing 1 | Animal | 1.08 | -0.008 |
| 2 | Positive | High | Dolphin Singing 2 | Animal | 0.975 | 0.094 |
| 3 | Positive | High | Crowd Cheering 1 | Human | 0.708 | 0.179 |
| 4 | Positive | High | Crowd Cheering 2 | Human | 0.595 | 0.522 |
| 5 | Positive | High | Rollercoaster 1 | Human | 0.769 | 0.295 |
| 6 | Positive | High | Rollercoaster 2 | Human | 0.687 | 0.507 |
| 7 | Positive | High | Speedboat 1 | Inanimate | 0.677 | 0.222 |
| 8 | Positive | High | Speedboat 2 | Inanimate | 0.782 | 0.229 |
| 9 | Positive | Low | Birds Singing 1 | Animal | 1 | -0.363 |
| 10 | Positive | Low | Birds Singing 2 | Animal | 0.92 | -0.579 |
| 11 | Positive | Low | Baby Cooing 1 | Human | 1.15 | -0.415 |
| 12 | Positive | Low | Baby Cooing 2 | Human | 1.037 | -0.052 |
| 13 | Positive | Low | Kids Playing 1 | Human | 1.075 | -0.152 |
| 14 | Positive | Low | Kids Playing 2 | Human | 1.026 | -0.028 |
| 15 | Positive | Low | Wind Chimes 1 | Inanimate | 0.659 | -0.742 |
| 16 | Positive | Low | Wind Chimes 2 | Inanimate | 0.829 | -0.766 |
| 17 | Negative | High | Monkeys Fighting 1 | Animal | -0.637 | 0.427 |
| 18 | Negative | High | Monkeys Fighting 2 | Animal | -0.56 | 0.563 |
| 19 | Negative | High | Riot 1 | Human | -1.212 | 0.383 |
| 20 | Negative | High | Riot 2 | Human | -1.106 | 0.442 |
| 21 | Negative | High | Men Fight 1 | Human | -1.017 | 0.279 |
| 22 | Negative | High | Men Fight 2 | Human | -1.114 | 0.226 |
| 23 | Negative | High | Ambulance 1 | Inanimate | -0.856 | 0.309 |
| 24 | Negative | High | Ambulance 2 | Inanimate | -0.824 | 0.38 |
| 25 | Negative | Low | Lions Fighting 1 | Animal | -0.881 | 0.131 |
| 26 | Negative | Low | Goose Fighting 2 | Animal | -0.771 | -0.014 |
| 27 | Negative | Low | Baby Crying 1 | Human | -0.877 | -0.22 |
| 28 | Negative | Low | Baby Crying 2 | Human | -1.001 | -0.223 |
| 29 | Negative | Low | Snoring | Human | -0.252 | -0.669 |
| 30 | Negative | Low | Coughing | Human | -0.954 | -0.288 |
| 31 | Negative | Low | Tsunami | Inanimate | -1.005 | -0.091 |
| 32 | Negative | Low | Toilet | Inanimate | -0.901 | -0.582 |

Note: Valence and arousal values are derived from multidimensional scaling solution from a separate group of participants (n=49).
