# Supplementary material for: Identifying Core Affect in Individuals from fMRI Responses to Dynamic Naturalistic Audiovisual Stimuli
Source: PLoS One. 2016 Sep 6;11(9):e0161589. doi: 10.1371/journal.pone.0161589 (PMC5012606; doi:10.1371/journal.pone.0161589)
Supplement: S2 Table — (DOCX) [file pone.0161589.s005.docx]

**Table S2. Summary of group analysis of functional localizer masks.**

|  |  |  | MNI coordinates | | |  |  |
| --- | --- | --- | --- | --- | --- | --- | --- |
| Anatomical region | Hemisphere | Cluster size | x | y | z | T | Z |
| VA∩(VP⋃AP)^c^ | | | | | | | |
| STG | R | 187 | 66 | -28 | 10 | 24.11 | 6.27 |
| Fusiform Gyrus | R | 512 | 27 | -61 | -5 | 21.06 | 6.06 |
| Middle Occipital Gyrus | L | 410 | -39 | -76 | 1 | 20.13 | 5.99 |
| Cuneus | R | 22 | 15 | -94 | 7 | 15.18 | 5.53 |
| Middle Occipital Gyrus | L | 21 | -15 | -88 | -8 | 14.26 | 5.48 |
| STG | L | 14 | -45 | -16 | -2 | 13.32 | 5.31 |
| Calcarine | L | 16 | -18 | -55 | 10 | 12.32 | 5.17 |
| STG | L | 19 | -45 | -34 | 13 | 11.64 | 5.07 |
| STG | L | 5 | -57 | -4 | 1 | 10.21 | 4.83 |
| VA | | | | | | | |
| Middle Occipital Gyrus | L | 321 | -36 | -76 | 7 | 24.73 | 6.31 |
| Inferior Occipital Gyrus | R | 528 | 36 | -82 | -5 | 23.23 | 6.22 |
| STG | R | 147 | 60 | -19 | 4 | 16.54 | 5.67 |
| Thalamus | R | 20 | 21 | -25 | -2 | 14.55 | 5.46 |
| Temporal Pole | R | 6 | 54 | 11 | -5 | 13.59 | 5.34 |
| Thalamus | L | 35 | -24 | -28 | 1 | 13.52 | 5.33 |
| STG | L | 17 | -45 | -34 | 16 | 13.45 | 5.32 |
| MTG | R | 9 | 54 | -40 | 13 | 13.09 | 5.28 |
| MTG | L | 7 | -51 | -34 | 4 | 11.83 | 5.10 |
| Lingual | L | 8 | -18 | -49 | -11 | 11.82 | 5.10 |
| Fusiform | L | 10 | -36 | -52 | -17 | 11.63 | 5.07 |
| Heschl Gyrus | L | 10 | -45 | -16 | 4 | 11.42 | 5.04 |
| STG | L | 6 | -57 | -7 | 1 | 11.16 | 5.00 |
| Middle Occipital Gyrus | R | 5 | 27 | -79 | 28 | 10.90 | 4.95 |
| Middle Occipital Gyrus | L | 6 | -27 | -76 | 31 | 10.79 | 4.93 |
| VP | | | | | | | |
| Middle Occipital Gyrus | L | 80 | -12 | -100 | 1 | 17.10 | 5.73 |
| Middle Occipital Gyrus | R | 156 | 33 | -91 | 13 | 15.94 | 5.61 |
| AP | | | | | | | |
| Heschl Gyrus | R | 12 | 60 | -22 | 10 | 14.58 | 5.46 |
| Heschl Gyrus | L | 7 | -42 | -31 | 13 | 11.95 | 5.12 |

R, right; L, left; cluster size indicates N voxels; T indicates peak t values; Z indicates peak z values; VA, voxels that were more responsive to audiovisual condition compared to baseline (*p* < .05, FWE-corrected, cluster size > 5); VP, voxels that were more responsive to checkerboard condition compared to baseline (*p* < .05, FWE-corrected, cluster size > 5); AP, voxels that were more responsive to beep condition compared to baseline (*p* < .05, FWE-corrected, cluster size > 5); VA∩(VP⋃AP)^c^: voxels that were more responsive to audiovisual condition compared to baseline (*p* < .05, FWE-corrected, cluster size > 5), but excluding those voxels that were more responsive to checkerboard condition compared to baseline (*p* < .05, FWE-corrected, cluster size > 5) and those voxels that were more responsive to beep condition compared to baseline (*p* < .05, FWE-corrected, cluster size > 5).
